# Serum-free adapted Drosophila S2R+ line is amenable to RNA interference

**DOI:** 10.17912/micropub.biology.000362

**Published:** 2021-01-29

**Authors:** Arthur Luhur, Daniel Mariyappa, Kristin M Klueg, Stephen L Rogers, Andrew C Zelhof

**Affiliations:** 1 Drosophila Genomics Resource Center; 2 Department of Biology; 3 Indiana University; 4 The University of North Carolina, Chapel Hill

## Abstract

We have previously adapted a select number of *Drosophila* cell lines to grow in serum-free media supplemented with fly extract. This condition is arguably more representative of a native growth environment. Here, we validated that the fly extract adapted line, S2R+ (FEx 2.5%) is amenable to RNAi. RNAi against *Rho1* in both S2R+ and S2R+ (FEx 2.5%) produced phenotypes similar to ones previously described in *Drosophila* S2 cells.

**Figure 1:**
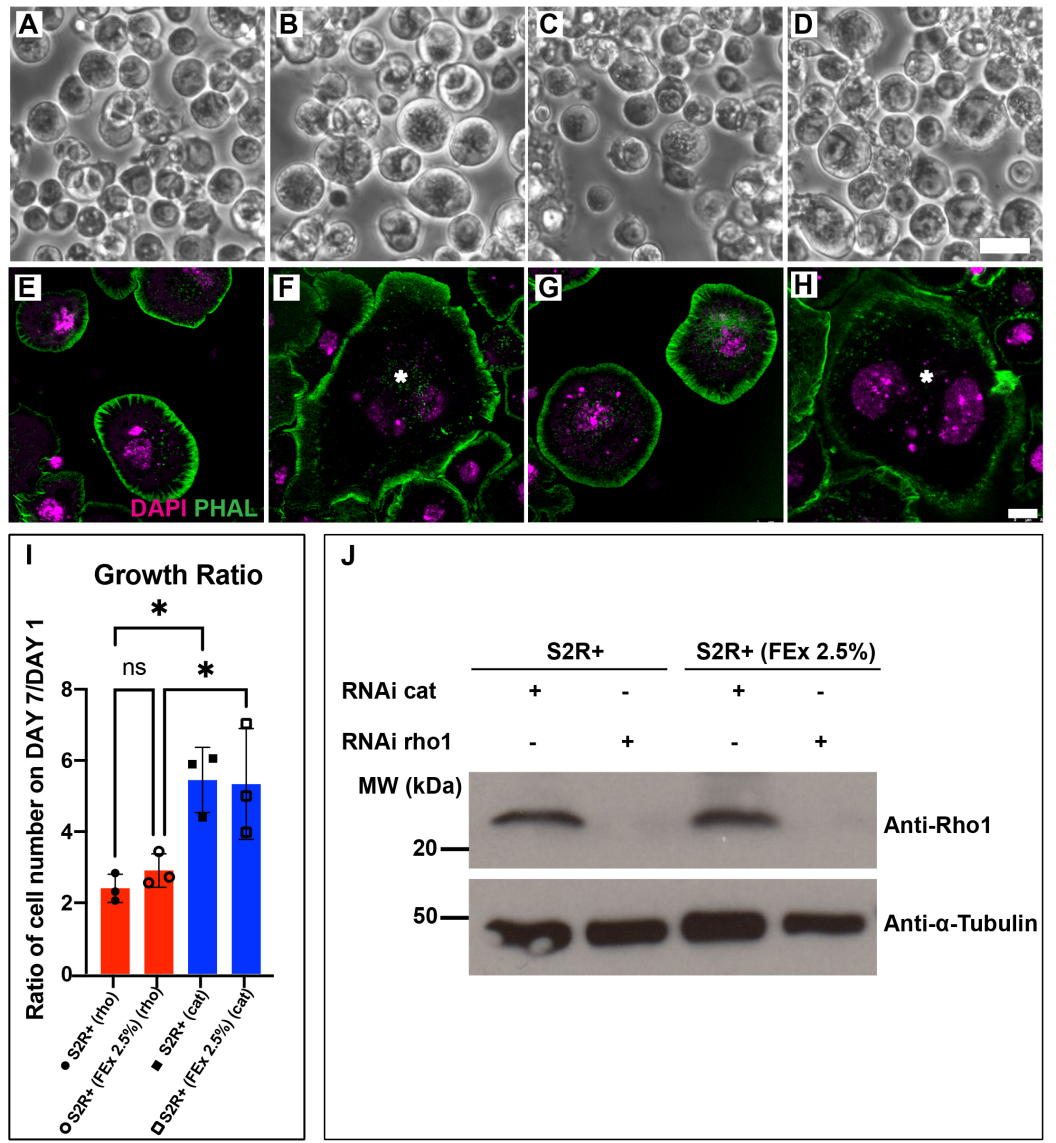
**A-D. Light micrographs of S2R+ and S2R+ (FEx 2.5 %) cells.** S2R+ cells cultured in M3 + BPYE + 10% FBS (A,B) were treated with either dsRNA against *cat* (A) or dsRNA against *Rho1* (B). S2R+ (FEx 2.5%) cells cultured in M3 + 2.5% FEx (C,D) were treated with either dsRNA against *cat* (C) or dsRNA against *Rho1* (D). Scale bar = 10 micrometers. **E-H Fluorescence confocal images of S2R+ cells and S2R+ (FEx 2.5%) cells**. S2R+ cells cultured in M3 + BPYE + 10% FBS (E,F) were treated with either dsRNA against *cat* (E) or dsRNA against *Rho1* (F). S2R+ (FEx 2.5%) cells cultured in M3 + 2.5% FEx (G,H) were treated with either dsRNA against *cat* (G) or dsRNA against *Rho1* (H). * marks an enlarged and multi-nucleated cell. DAPI (purple) marks the DNA and phalloidin (green) marks cytoskeletal actin. Scale bar = 8 micrometers. **I.** A bar graph shows the ratio of cell numbers after seven days of RNAi treatment against *Rho1* or *cat*, to the cell number on day 0. * denotes p < 0.05. **J**. Immunoblot of Rho1 and α-Tubulin from S2R+ and S2R+ (FEx 2.5%) cells, and treated with dsRNA against *Rho1* or dsRNA against *cat*. α-tubulin serves as loading control.

## Description

*Drosophila* Schneider S2 cell lines are susceptible to RNA interference (RNAi) and this attribute has cemented *Drosophila* cell lines as an important tool for high throughput functional genomics screening (Rogers and Rogers 2008; Zhou *et al.* 2013; Mohr 2014). RNAi against Drosophila *Rho1* in S2 cells results in a block in mitosis, giving rise to enlarged and multinucleated cells (Rogers *et al.* 2004). Recently, we have adapted a select group of *Drosophila* embryonic cell lines to grow in media supplemented by adult fly extract (FEx), instead of fetal bovine serum (FBS) (Luhur *et al.* 2020). Here, we demonstrate that S2R+ (FEx 2.5%), the M3 + 2.5% FEx-adapted S2R+ line is also amenable to RNA interference (RNAi), similar to its parental S2R+ cells cultured in M3 BPYE + 10% FBS. We observed similar efficacious RNAi against *Rho1* in S2R+ and S2R+ (FEx 2.5%) as the cells became enlarged (Figure 1A-D), multinucleated (Figure 1E-H) and failed to proliferate (Figure 1I). There was a comparable growth delay in S2R+ and S2R+ (FEx 2.5%) cells treated with *Rho1* dsRNA, as the cell population doubled in 7 days (Figure 1I). In contrast, both S2R+ and S2R+ (FEx 2.5%) cells treated with double stranded RNA against a control target gene encoding the bacterial antibiotic resistance gene *chloramphenicol acetyl transferase* (*cat*) had significantly proliferated 5 fold more under similar conditions (Figure 1I). In addition, there were no significant differences in the growth ratio between S2R+ and S2R+ (FEx 2.5%) (Figure 1I) (Luhur *et al.* 2020). These results demonstrate that *Rho1* RNAi was recapitulated robustly in S2R+ (FEx 2.5%), similar to its parental S2R+ cells. Lastly, to confirm the depletion of *Rho1*, we assayed for Rho1 protein levels in these cultures by Western blot. Our result indicated a strong reduction in the amount of Rho1 protein in both S2R+ (FEx 2.5%) and S2R+ cells after *Rho1* knockdown (Figure 1J). In contrast, the control RNAi knockdown of *cat* did not affect Rho1 protein levels in either S2R+ or S2R+ (FEx 2.5%) (Figure 1J). In summary, this finding expands the utility of the fly extract-adapted cells for their use in functional genomics in a more physiologically relevant culture condition.

## Methods

Cell culture

S2R+ (DGRC#150, FBtc0000150), S2R+ (FEx 2.5%) (DGRC#310, FBtc0000310) were cultured in M3 + BPYE + 10% FBS and M3 + 2.5% FEx, respectively, according to previously described protocol (Luhur *et al.* 2019).

RNA interference (RNAi)

Cells from the respective growth media were pelleted and then seeded at 1 million cells/ mL in serum free M3 media in a 24 well plate (1 mL per well). 10 mg/mL dsRNA was added slowly to the media and allowed to incubate at room temperature for 1 hour. After the incubation period, the M3 media was supplemented with equal volumes of either M3+BPYE+20% FBS or M3 + 5% FEx, to constitute the M3 + BPYE + 10% FBS and M3 + 2.5% FEx, respectively. Cells were allowed to grow for a week at 25°C before assaying for the loss of function phenotypes.

As a negative control, a 467-bp fragment of the chloramphenicol resistance cassette was amplified from pFastBacHT-CAT expression plasmid (Invitrogen) using the primers: T7-CAT-fwd: 5′-TAATACGACTCACTATAGGATCCCAATGGCATCGTAAAGAACATTTTGAGGC-3′ and T7-CAT-rev: 5′-TAATACGACTCACTATAGGGGGCGAAGAAGTTGTCCATATTGGCCA-3′.

As a positive control, we amplified a 667-bp sequence for *Rho1*(FBgn0014020) using the primers: T7-Rho1-fwd: 5′-TAATACGACTCACTATAGGTTTGTTTTGTGTTTAGTTCGGC-3′ and T7-Rho1-rev: 5′-TAATACGACTCACTATAGGATCAAGAACAACCAGAACATCG-3′, from a Rho1 expression construct, originally provided by Dr. Liqun Luo (Stanford University). The dsRNA synthesis protocols followed the protocol described (Rogers and Rogers 2008).

Immunostaining and microscopy

The cells were seeded on dishes coated with Concanavalin A. After one hour, the media was removed and the cells were fixed for 10 minutes in a solution containing 4% paraformaldehyde diluted in phosphate buffered saline (PBS). The cells were then rinsed in 0.1% PBS-Triton-X and incubated in 1:1000 phalloidin for two hours at room temperature. Subsequently, the cells were rinsed for three times in 0.1% PBS-Triton-X before being mounted on Vectashield mounting media containing DAPI (H-1300). Fluorescence imaging was carried out using the Leica SP8 confocal microscope.

Protein extraction and Western Blotting

Cell pellets from a single well of a 24-well plate cells subjected to RNAi against either *Rho1* or *cat* (dsRNA control) were lysed with RIPA buffer. Proteins in the lysed samples were separated with a BioRad WGX 4-20% gel, transferred onto Nitrocellulose membrane (BioRad) and blotted with either mouse anti-Rho1 (p1D9, from Developmental Studies Hybridoma Bank deposited by Parkhurst, S) or mouse anti- α-tubulin (T9026, Sigma). The blots were treated with anti-mouse HRP and the signals were visualized using Pierce Enhanced Chemiluminescence Reagent (ThermoFisher). The experiment was conducted in duplicate.

Cell counting and statistical analysis

Live cells were counted using an automated cell counter (BIORAD) according to manufacturer’s instructions. Each condition had a total of three replicate counts. Statistical analysis of the differences in the growth ratio was carried out using Prism8 using ordinary-one way ANOVA test, with Sidak’s multiple comparison test.
